# MicroRNA expression and activity in T-cell acute lymphoblastic leukemia

**DOI:** 10.18632/oncotarget.23539

**Published:** 2017-12-20

**Authors:** Fang Ye

**Affiliations:** ^1^ Department of Hematology, Beijing Chuiyangliu Hospital Affiliated to Tsinghua University, Beijing, China

**Keywords:** MicroRNA, T-ALL, NOTCH1

## Abstract

T-cell acute lymphoblastic leukemia (T-ALL) is a lymphoid malignancy caused by the oncogenic transformation of immature T-cell progenitors. Many biologically relevant genetic and epigenetic alterations have been identified as driving factors for this transformation. Recently, microRNAs (miRNAs) have been shown to influence various leukemias, including T-ALL. Aberrant expression of miRNAs can function as either oncogenes or tumor suppressors in T-ALL through the regulation of cell migration, invasion, proliferation, apoptosis, and chemoresistance. This occurs by targeting key signaling pathways or transcriptional factors that play a critical role in T-ALL pathology and progression. Different miRNA expression profiles have been linked to specific genetic subtypes of human T-ALL. Furthermore, miRNAs can also act as independent prognostic factors to predict clinical outcomes for T-ALL patients. In the current review, we will focus on the role of miRNAs in the development and progression of T-ALL.

## INTRODUCTION

T-cell acute lymphoblastic leukemia (T-ALL) is a lymphoid malignancy caused by oncogenic transformation of immature T-cell progenitors [1]. This aggressive hematologic malignancy is typically characterized by diffuse infiltration of the bone marrow by malignant lymphoblasts expressing immature T-cell surface markers. Currently, T-ALL accounts for 20−25% of adult and 10−15% of pediatric cases of ALL [2, 3]. Clinically, patients with T-ALL typically present with high white cell counts in the blood. Patient symptoms include: hematopoiesis inhibition with anaemia, pyrexia, thrombocytopenia, hemorrhage, infection, frequent large thymic masses, as well as pleural, and meningeal infiltration at diagnosis. Abnormal genetic or epigenetic alterations can lead to an arrest of differentiation and uncontrolled expansions of immature thymocytes with distinct gene expression patterns during T-cell development. These alterations end up, resulting in the transformation of normal T-cells into fully transformed T-ALL lymphoblasts [4]. T-ALL can be classified into different clinically relevant biological subgroups according to unique gene expression signatures [5, 6] and distinct immunophenotypes that reflect immature thymocytes arresting at different stages of differentiation [7]. Generally, T-ALL can be classified in three categories. Early T-cell precursor acute lymphoblastic leukemia (ETP-ALL) is blocked at the earliest stages of T-cell differentiation with a distinctive immunophenotype (CD1a^−^CD8^−^CD5^weak^) and presenting stem-cell/myeloid markers [8]. ETP-ALL has a lower frequency of *NOTCH1* mutations and homozygous cyclin-dependent kinase inhibitor 2A/2B (*CDKN2A*/*CDKN2B*) deletions. Genetically it is characterized by mutations in genes regulating cytokine receptors and signaling that disrupts hematopoietic development, histone-modifying genes, and epigenetic regulators [9, 10]. In contrast, the other two subgroups of T-ALL frequently show *NOTCH1* mutations and homozygous *CDKN2A*/*CDKN2B* deletions. A second subgroup of T-ALLs with a CD1a^+^CD4^+^CD8^+^ immunophenotype has an excellent prognosis related to the early stages of cortical thymocyte maturation [11]. This subgroup typically shows activation of the T-cell leukemia homeobox 1 (*TLX1*/*HOX11*), T-cell leukemia homeobox 3 (*TLX3*/*HOX11L2*), NK2 homeobox 1 (*NKX2-1*) and NK2 homeobox 2 (*NKX2-2*) genes [5, 12]. Another subgroup of T-ALLs correlated with a late cortical thymocyte immunophenotype (CD4^+^CD8^+^CD3^high^) typically display activation of the T-cell acute lymphocytic leukemia 1/stem cell leukemia (*TAL1*/*SCL*) oncogene [5].

T-ALL arises as result of a multistep oncogenic process. Many biologically relevant genetic and epigenetic alterations have been identified as driving events for this process. These events coordinately disrupt key oncogenic, tumor suppressive and developmental pathways responsible for the normal control of cell growth, proliferation, motility, survival and differentiation during thymocyte development. One of the key regulators in early T-cell development and T-ALL oncogenic signals is *NOTCH1*, which is constitutively active in more than 50% of T-ALLs [13]. *NOTCH1*-activating mutations frequently co-occur with loss of the *CDKN2A* locus [9]. Together these result in the aberrant expression of various T-cell fate specific and thymocyte development related transcription factors and epigenetic regulators that can become oncogenes or tumor suppressors. Mutated-*NOTCH1* forms regulatory networks with these transcription factors and epigenetic regulators contributing to T-ALL development [1, 14].

Currently, intensified chemotherapy in T-ALL treatment results in a favorable overall survival for patients with 80% in children [15] and 60% in adults [16]. However, the concomitant side effects on the central nervous system and bone development should not be underestimated [17]. The prognosis of T-ALL patients with primary resistance who fail to achieve complete remission or those suffering from relapses after an initial treatment remains poor [18], with a recurrence rate of up to 20% in pediatric and 40% in adult patients [15]. A better understanding of the molecular mechanisms of T-ALL needed to identify more specific targets and develop less toxic anti-leukemic drugs. Over the past few decades, great progress has been made in understanding of the genetics and biology of T-ALL, especially on protein coding genes. More recently, through the study of mutations in the noncoding regions of the genome that lead to aberrant transcription factor expression in T-ALL, a number of microRNAs (miRNAs) were identified. These miRNAs have been linked with these genes and play an important role in pathology of T-ALL [19].

MiRNAs were first discovered in 1993 through the analysis of developmental timing mutants in *C. elegans* [20, 21]. Since then, over 2,500 miRNAs have been discovered so far in the human genome (http://www.mirbase.org). They are an endogenous, non-coding RNA, composed of 22–24 nucleotides (nt), which function as post-transcriptional gene regulators [22]. MiRNA biogenesis starts with long primary miRNA (pri-miRNA, 60-80nt) transcripts containing one or more hairpin structures produced by RNA polymerase II or III in the nucleus. They are then further processed into a smaller stem-loop (approximately 70 nt) precursor miRNA (pre-miRNA) by RNase III-type endonuclease Drosha. The pre-miRNA is then transported to the cytoplasm by Exportin 5 and is cleaved into a mature double stranded miRNA molecule (approximately 22 nt) by the Dicer ribonuclease. The guide strand is then incorporated into the RNA-induced silencing complex (RISC), guiding RISC to its target messenger RNA (mRNA) through complementary base pairing on the 3′ untranslated region (3′UTR) of the target mRNA. This pairing and binding to the target mRNA leads to mRNA degradation or translation repression [23, 24]. One third of protein encoding genes are regulated by miRNAs, including genes involved in physiological processes [25, 26] as well as with the pathological development and progression of cancer [27, 28]. Recently, miRNAs have been shown to influence various leukemias, including T-ALL. Aberrant expression of miRNAs can function as either oncogenes or tumor suppressors in T-ALL. MiRNAs through the regulation of cell migration, invasion, proliferation, apoptosis, and chemoresistance by targeting key signaling pathways or transcriptional factors play a critical role in T-ALL pathology and progression [19, 29, 30]. Additionally, different miRNAs expression profiles have been linked to specific genetic subtypes of human T-ALL [31, 32]. Furthermore, miRNAs can also act as independent prognostic factors to predict clinical outcomes for T-ALL patients [33]. In the current review, we will focus on the role of miRNAs in the development and progression of T-ALL.

### MiRNAs are differentially expressed in T-ALL subpopulation

T-ALLs can be classified into different genetic subtypes according to unique protein coding gene expression signatures and immunophenotypes as described above [5, 8, 11]. Consistently, studies have found that different genetic subgroups of T-ALL also had subtype-specific miRNA expression profiles. Research by Nagel *et al*. [34] first demonstrated that the miR-17-92 cluster was overexpressed in cell lines and primary cells that were positive for the expression of *TLX1*, *TLX3* and NK2 homeobox 2-5 (*NKX2-5*). These are members of the NK-like family of homeobox genes which are ectopically activated in the subtype of T-ALL cells with a CD1a^+^CD4^+^CD8^+^ immunophenotype [5, 12]. This miRNA cluster was regulated by these transcription factors leading to the enhanced survival of leukemic T-cells though decreasing E2F transcription factor 1 (E2F1) protein expression [34]. MiR-223 was found overexpressed in a subset of myeloid-like adult T-ALLs which appeared to have an unfavorable clinical outcome [31].

Aberrant expression of homeobox A (HOXA) genes are one of the diverse characteristics of T-cell-specific transcription factors that can function as oncogenes [6]. High expression of miR-196b specifically occurred in MLL-rearranged and T-ALL patients carrying *CALM-AF10* or *SET-NUP214* fusions and inversion of chromosome 7. These molecular and morphological changes are functionally linked with upregulation of *HOXA*. Since miR-196b is encoded on the *HOXA* cluster, miR-196b and *HOXA* genes might be co-activated in acute lymphoblastic leukemia [35]. In addition, miR-196a and miR-196b expression was associated with an immature (IMM) immunophenotype and expression of CD34 and CD33, with both targeting ETS transcription factor *ERG* in T-ALL patients [36].

Furthermore, Coskun *et al*. [32] identified a set of miRNAs that were differently expressed in adult ETP-ALL by comparing miRNA profiles between ETP-ALL and non-ETP T-ALL patients. Their results included two of the most upregulated (miR-221 and miR-222) and six downregulated miRNAs (miR-151-3p, miR-19a, miR-20b, miR-342-3p, miR-363, and miR-576-3p). It was also discovered that miR-222 inhibited proliferation, caused cell cycle arrest and apoptosis in leukemic cells by directly inhibiting the expression of the proto-oncogene *ETS1* through *in vitro* studies, which has been shown to be concomitant with the poor prognosis of ETP-ALL.

These data suggest that miRNAs are differentially expressed in subpopulations of T-ALL as genetic alterations and the illumination of subtype-specific miRNA expression profiling could contribute to better diagnosis and treatment for T-ALL patients. However, more comprehensive studies remain to be reported.

### MiRNAs expression and signaling pathways in T-ALL

### Oncogenic NOTCH1 signal in T-ALL

The NOTCH1 signaling pathway is prominent for early T-cell fate specification, which is the process by which multipotent hematopoietic progenitors commit to T-cell lineage, as well as further thymocyte development [37, 38]. Aberrant, constitutively active NOTCH1 signaling is the predominant oncogenic event involved in the pathogenesis of T-ALL [13]. NOTCH1 is a class I transmembrane glycoprotein composed of functional extracellular (NECD), transmembrane (TM), and intracellular (NICD) domains. NOTCH1 functions as a receptor for membrane-bound ligands Jagged1, Jagged2 and Delta1, in order to regulate cell-fate determination. Upon ligand activation, the NICD is released through cleavage by a member of a disintegrin and metalloproteinase (ADAM) family of proteases and the gamma secretase complex, and then translocates to the nucleus. In the nucleus, NICD binds to the CBF-1/suppressor of hairless/Lag-1 (CSL) transcription factor complex, resulting in the subsequent activation of the canonical Notch target genes [39]. Many canonical downstream targets that mediate oncogenic activity of NOTCH1 in T-ALL have been described [1, 40]. Recently, it has become increasingly evident that alterations in the expression of miRNAs also play an important role in the NOTCH1 regulatory network during T-ALL progression.

### NOTCH1-driven T-ALL mouse model

The oncogenic role of NOTCH1 in T-ALL can be illustrated by the rapid development of acute leukemia in murine models. Currently a mouse model is being used that transplants hematopoietic progenitors with a constitutively active, intracellular form of NOTCH1 [41]. Much progress has been made in elucidation of the oncogenic or tumor suppressor role of miRNAs in NOTCH1-driven T-ALL with this model. To establish this model, fetal liver cells with hematopoietic progenitor cells (HPCs) isolated from pregnant mice were retrovirally transduced with an intracellular NOTCH1 (ICN1) construct as well as a construct encoding antagomiR or premiR. These HPCs were subsequently injected into the tail vein of lethally irradiated recipient mice [41, 42]. This model has also been modified where, the transduced HPCs can also be directly injected into irradiated syngeneic mice via the tail vein [43]. The T-ALL onset was then monitored and the miRNA profiles could be used to identify potential oncogenic or tumor suppressive miRNAs as compared with control mice.

Konstantinos *et al*. [44] demonstrated that a novel translocation targeting the miR-17-92 cluster coincided with a second rearrangement that activates *NOTCH1* in T-ALL. Among these are miR-19, which showed the highest expression of all members of the miR-17-92 cluster in human T-ALL. This expression of miR-19 is sufficient enough to promote leukemogenesis in NOTCH1-induced T-ALL *in* *vivo* through coordination a phosphatidylinositol-3-OH kinase (PI3K) pathway related program of cell survival. The PI3K pathway related program directly targets its components including *BIM* (*BCL2L11*), AMP-activated kinase (*PRKAA1*), and tumor suppressor phosphatases *PTEN* and *PP2A* (*PPP2R5E*). These observations indicated a collaborative role of *NOTCH1* and the miR-17-92 cluster in T-ALL development. Moreover, four miRNAs in the miR-17-92 cluster and four important genes (*CYLD*, *HOXA9*, *BCL2L11* and *RUNX1*) were found in a “miRNAs and genes co-regulatory network” by a target prediction algorithms assay of genes and miRNAs known to be involved in T-ALL via bioinformatics [45]. MiR-19 was confirmed to regulate NF-κB signaling through direct targeting of *CYLD* with further *in vitro* experiments [45]. Mavrakis *et al*. [46] further identified five miRNAs (miR-19b, miR-20a, miR-26a, miR-92 and miR-223) that contributed to leukemogenesis and acted as multi-targeted regulators of several tumor suppressor genes (*IKAROS*/*IKZF1*, *PTEN*, *BIM*, *PHF6*, *NF1* and *FBXW7*). The Mavrakis's research discovered these miRNAs through cross-comparison of miRNA expression profiles in human T-ALL with an unbiased genetic screen and computational analyses. Three of these miRNAs (miR-19b, miR-20a, and miR-92) belong to the oncogenic miR-17-92 cluster. All five miRNAs could promote T-ALL development in a NOTCH1-driven mouse model. These miRNAs had overlapping and cooperative effects on tumor suppressor genes with miR-19b directly targeting *PTEN* and *BIM*; miR-20a directly targeting *PTEN*, *BIM* and *PHF6*; miR-92 directly targeting *IKAROS*/*IKZF1*, *PTEN*, *BIM*, *NF1* and *FBXW7*, and miR-223 directly targeting *FBXW7*, respectively. MiR-223 was subsequently shown to be activated by *TAL1* [47] and *NOTCH1* [48], two important T-ALL oncogenes. Mets *et al*. further investigated miRNAs that directly target the tumor suppressor PHF6 by performing an unbiased *PHF6* 3′UTR-microRNA library screen and combined the results with microRNA profiling data of samples from patients with T-ALL. MiR-128-3p was selected as a candidate for PHF6-targeting, which could significantly accelerate leukemia onset in a NOTCH1-induced T-ALL mouse model upon over-expression [49]. In addition, another study demonstrated that Dicer1-mediated miRNA biogenesis was essential for development, progression, and maintenance of NOTCH1-driven T-ALL in leukemic mice, which is a key component of the miRNA processing machinery [50]. With conditional knockout, the biallelic loss of Dicer1 resulted in the abrogation of established NOTCH-induced T-ALLs, indicating that the NOTCH-on transformed state is dependent on one or more miRNAs. They further delineated that miR-21 promoted survival of T-ALL cells, in part through the repression of the tumor suppressor gene programmed cell death 4 (*PDCD4*) in NOTCH-addicted murine and human T-ALL cell lines based on miRNA expression profile data and *in vitro* experiments.

Many tumor-suppressive miRNAs have also been identified with NOTCH1-sensitized T-ALL mouse model. Sanghvi *et al*. characterized a set of functionally interconnected tumor-suppressive miRNAs with this model [43]. First, they screened miRNAs that exhibited decreased expression by at least 10-fold in primary T-ALL samples as compared to normal tissues. They then performed functional assessments by *in vitro* proliferation assays and *in vivo* miRNA loss-of-function studies evaluating their leukemogenesis effects. They accomplished this in a murine, NOTCH1-driven, T-ALL model with miRNAs knockout, miRNA sponges or lentiviral miRZips approaches. After selection, five miRNAs were characterized (miR-29, miR-31, miR-150, miR-155, and miR-200), all with tumor-suppressive effects in NOTCH1-driven T-ALL model. Using a computational strategy to eliminate genes that were exclusively targeted by the tumor-suppressive miRNAs, they identified the known T-ALL oncogene *MYB* (target of miR-150, miR-155, and miR-200). They also discovered a potential new oncogene in T-ALL, *HBP1* (target of miR-29, miR-31, miR-155, and miR-200), which encodes a transcription factor. Their research then focused on these genes as key targets of this tumor-suppressive miRNA network. The expression of both genes was increased in T-ALL patient samples, and each gene promoted the progression of T-ALL in mice. Interestingly, it was also noted that the oncogenic NOTCH1/MYC pathway inhibited miR-31, miR-150, and miR-155 expression. This suggests that repression of the expression of a subset of tumor suppressor miRNAs in T-ALL cells might be a result of *NOTCH* and *MYC* activation. Mets *et al*. [42] focused on the oncogene *MYB* with post-transcriptional regulation by miRNAs in T-ALL through an unbiased, high-throughput dual luciferase-based *MYB* 3′UTR-miRNA library screen. They employed subsequent analyses of miRNA-mRNA correlation data during normal T-cell differentiation and in primary T-ALL patient samples. Followed by *in vitro* luciferase reporter assay, miR-193b-3p was detected as a direct negative regulator of *MYB*. Remarkably, downregulation of miR-193b-3p by antagomiR in the NOTCH1-induced mouse model significantly accelerated leukemia onset *in vivo*, suggesting the inhibition of tumor-suppressor miR-193b-3p can cooperate with NOTCH1 in T-ALL pathogenesis.

### MiRNAs directly involved in NOTCH1 oncogenic signaling in T-ALL

In NOTCH1-dependent T-ALL models, sustained NOTCH1-receptor signaling activates a broad array of genes involved in anabolic pathways, among which NOTCH1/MYC regulatory axis plays a major role. This results in NOTCH1-induced transformation through a feed-forward loop transcriptional circuitry [51]. This critical feed-forward loop regulatory network can be directly regulated by miRNAs [52, 53]. MiR-451 and miR-709 have been shown to modulate *MYC* expression and also act as downstream targets of NOTCH1 in murine T-ALL [52]. In this study, activation of NOTCH1 signaling by induction of ICN1 led to repression of miR-451 and miR-709 in an established mouse model of T-ALL. These repression effects were mediated by degradation of the E2A tumor suppressor followed by ICN1 induction, which transcriptionally activates the genes encoding miR-451 and miR-709. Importantly, both miR-451 and miR-709 directly repressed *MYC* expression and miR-709 also directly targeted the *AKT* and *Ras-GRF1* oncogenes. It was also demonstrated that decreased expression of miR-451 and miR-709 was necessary for initiation and maintenance of mouse T-ALL. Thus, miR-451 and miR-709 functioned as suppressors of oncogenesis within the NOTCH1/MYC regulatory axis of murine T-ALL. The NOTCH1/miR-451/MYC axis also played a part in human T-ALL (only miR-451 has human homologue). Moreover, MYC could also modulate NOTCH1 expression through a feed-forward loop via a miRNA intermediary [53]. The expression level of miR-30a, a member of a family of miRNAs that are transcriptionally suppressed by MYC [54], was negatively correlated with *NOTCH1* mutational status in T-ALL patient samples. Sustained NOTCH1 signaling upregulated *MYC* expression which resulted in decreased expression of miR-30a. MiR-30a could directly bind to 3′UTR of *NOTCH1* and inhibit *NOTCH1* expression. These discoveries depict a MYC/miR-30a/NOTCH1 feedback regulatory loop that by MYC-mediated inhibition of miR-30a de-repressed NOTCH1, eventually increasing its own expression [53]. Another miRNA that directly targeted NOTCH1 was miR-101, which was downregulated in T-ALL patient specimens and T-ALL cell lines [55]. MiR-101 acts as a tumor suppressor through the repression of proliferation and invasion, induction of apoptosis, and enhancement of chemotherapeutic sensitivity in T-ALL cells *in vitro* mediated by inhibiting NOTCH1 [19]. As discussed above, it was also shown that the oncogenic NOTCH1/MYC pathway inhibited the expression of tumor suppressor miRNAs, including miR-31, miR-150, and miR-155 [43]. Thus, NOTCH, MYC, and miRNAs formed a complicated and very important feedback regulatory network with each other which contributed to pathogenesis of T-ALL.

MiR-223 has been found to be overexpressed in a subset of T-ALLs and directly targeted *FBXW7*, a known tumor suppressor gene in T-ALL [31, 46]. Further studies by putative promoter analysis, luciferase and chromatin immunoprecipitation (ChIP) assays revealed that both NOTCH and NF-κB signal could directly and cooperatively activate the transcriptional activity of miR-223 promoter. Moreover, it also confirmed the negative regulation of *FBXW7* by miR-223 [48]. Similarly, another group [56] identified that deletion of miR-181ab1 expression inhibited the development of NOTCH1-induced T-ALL in mouse models and human cells through direct targeting of NOTCH regulated ankyrin repeat protein (*NRARP*), a negative regulator of NOTCH1 downstream signaling. Intriguingly, miR-181ab1 could control the strength and threshold of NOTCH activity in tumorigenesis where the miR-181ab1 deletion appeared to have stronger inhibitory effects on T-ALL cells with lower levels of ICN1 expression. It was also shown that miR-181ab1 could be specifically targeted to inhibit tumor development with little toxicity to normal development, in that the effects of miR-181ab1 deletion were compensated for during normal thymic progenitor development but not during T-ALL development [56].

**Figure 1:**
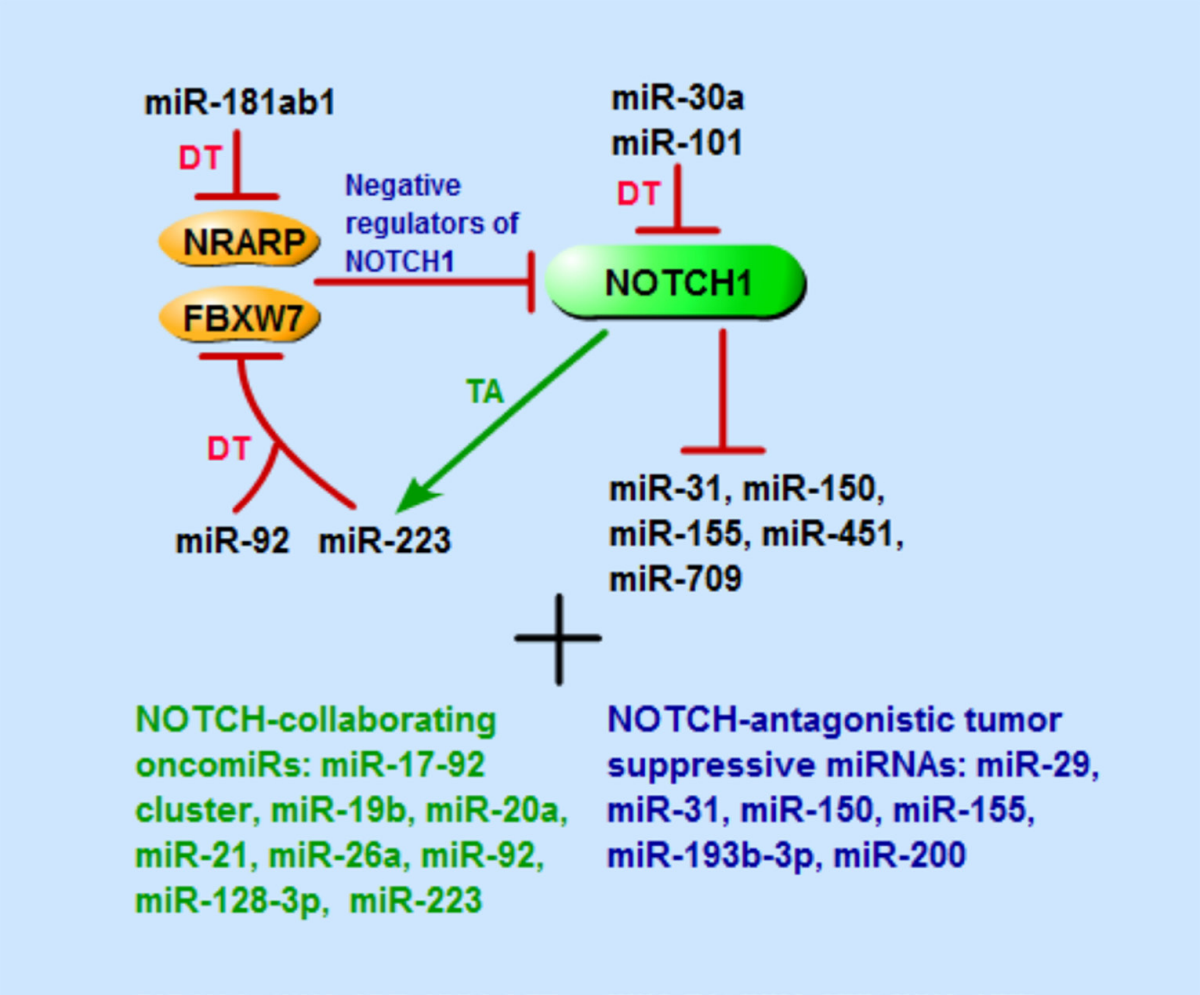
Schematic representation of miRNAs that are involved in NOTCH1-driven T-ALL, including miRNAs directly target or be targeted by NOTCH1 signaling pathway components, and that have collaborating or antagonistic effects with NOTCH pathway TA, transcriptional activation; DT, direct targeting.

**Figure 2:**
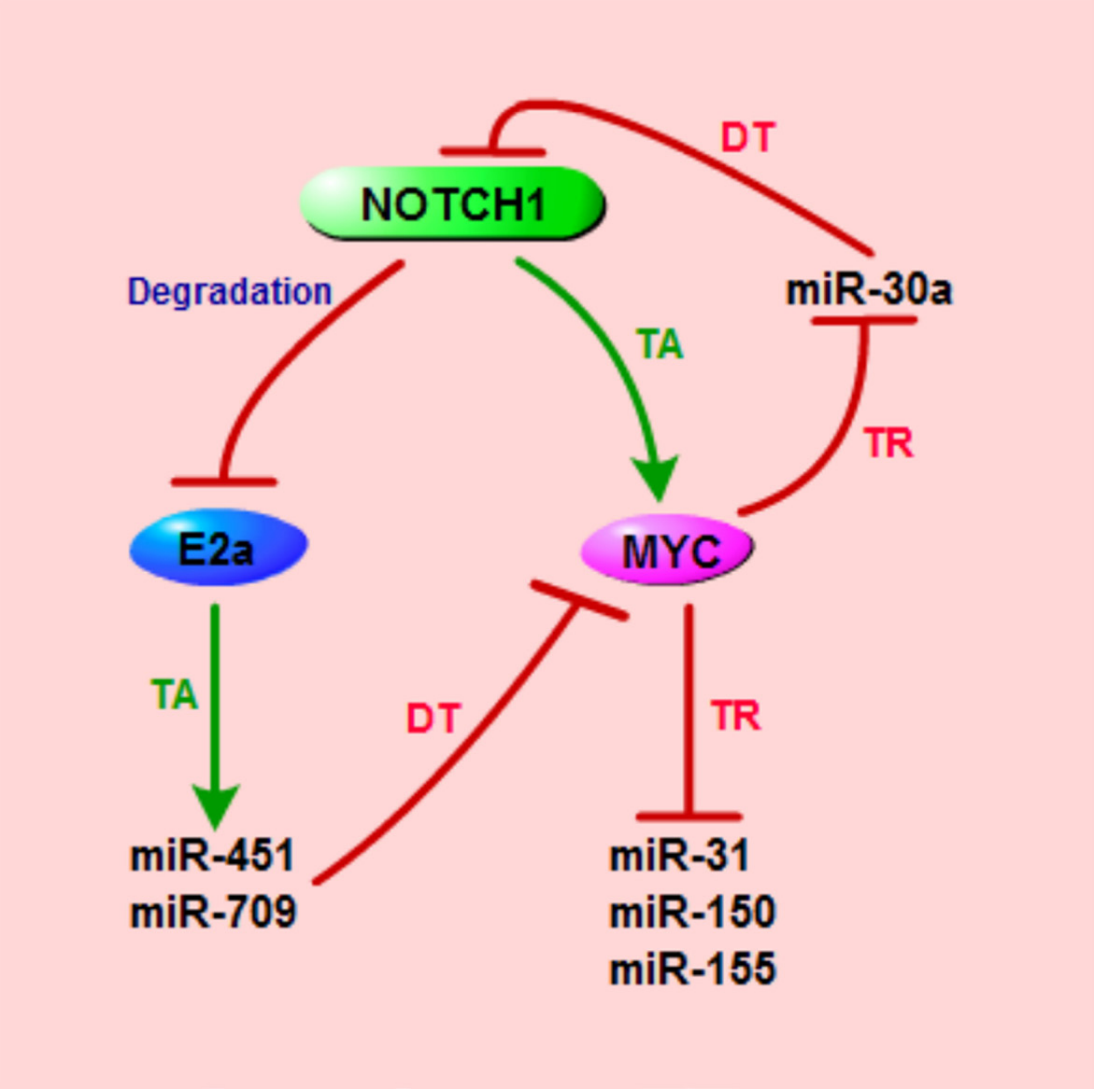
Schematic representation of miRNAs that are implicated in NOTCH1/MYC axis in T-ALL TA, transcriptional activation; TR, transcriptional repression; DT, direct targeting.

An overview of the described miRNAs involved in NOTCH-driven T-ALL and in NOTCH1/MYC axis can be found in Figures 1, 2 and in Table 1.

**Table 1:** miRNAs involved in T-ALL biology

miRNAs in/with	Deregulation	Direct targets	Function	References
**Pathways**				
**NOTCH**				
miR-30a	downregulation	NOTCH1	MYC repressed; forms a MYC/miR-30a/NOTCH1 feed-forward regulatory loop in NOTCH-driven T-ALL; represses growth; promotes apoptosis	[53]
miR-31	downregulation	HBP1	tumor suppressor in NOTCH1-driven T-ALL model; NOTCH1/MYC pathway repressed	[43]
miR-92	upregulation	FBXW7	oncomiR; induces NOTCH1 expression	[46]
miR-101	downregulation	NOTCH1	represses proliferation and invasion; induces apoptosis; enhances chemotherapeutic sensitivity	[19]
miR-150	downregulation	MYB	tumor suppressor in NOTCH1-driven T-ALL model; NOTCH1/MYC pathway repressed	[43]
miR-155	downregulation	MYB, HBP1	tumor suppressor in NOTCH1-driven T-ALL model; NOTCH1/MYC pathway repressed	[43]
miR-181ab1	upregulation	NRARP	controls the activity of NOTCH in tumorigenesis of NOTCH1-driven T-ALL	[56]
miR-223	upregulation	FBXW7	myeloid-like high; NOTCH1 activated; induces NOTCH1 expression; promotes growth	[31, 46–48]
miR-451	downregulation	MYC	NOTCH1 repressed; tumor suppressor among NOTCH1/MYC regulatory axis of mouse and human T-ALL	[52]
miR-709	downregulation	MYC, AKT, Ras-GRF1	NOTCH1 repressed; tumor suppressor among NOTCH1/MYC regulatory axis of mouse T-ALL	[52]
**PI3K/AKT**				
miR-19	upregulation	PP2A, PRKAA1, BIM, PTEN	increases phosphorylation of AKT and the ribosomal S6 protein; promotes survival	[44]
**NF-κB**				
miR-19	upregulation	CYLD	induces downstream of NF-κB;	[45]
miR-181a	upregulation	−	induces chemoresistance through activating AKT	[59]
miR-223	upregulation	FBXW7	NF-κB activated	[46–48]
**cAMP/PKA**				
miR-142-3p	upregulation	GRa, cAMP/PKA	promotes growth; induces glucocorticoid resistance; correlates with poor prognosis	[57]
**JAK/STAT**				
miR-21	upregulation	−	inhibits STAT3 protein expression, promotes proliferation and invasion; decreases apoptosis	[58]
**Transcription factors**				
**TAL1**				
miR-101	downregulation	TAL1	targets TAL1	[55]
miR-140-5p	downregulation	TAL1	targets TAL1	[55]
miR-146b-5p	downregulation	−	TAL1 repressed; inhibits migration and delays T-ALL progression	[63]
miR-223	upregulation	FBXW7	TAL1 repressed; mediates TAL1-induced growth	[47]
miR-448	downregulation	TAL1	targets TAL1	[55]
miR-485-5p	downregulation	TAL1	targets TAL1	[55]
miR-520d-5p	−	TAL1	targets TAL1	[55]
**HOXA**				
miR-196a	HOXA high	ERG	associated with an IMM and expression of CD34 and CD33	[36]
miR-196b	HOXA and IMM high	ERG	co-activated with HOXA; associated with an IMM and expression of CD34 and CD33	[36]
**SOX4**				
miR-204	downregulation	SOX4	inhibits proliferation and metastasis	[64]
**JunB**				
miR-149^*^	upregulation	JunB	promotes proliferation and suppresses apoptosis	[30]
**EGR1**				
miR-181a	upregulation	EGR1	enhances proliferation	[66]
**Other oncogenes or tumor suppressors**				
miR-16	−	−	negatively correlates with DFS and overall survival of childhood T-ALL patients	[82]
miR-19a	ETP-ALL low	−	ETP-ALL low	[32]
miR-19b	upregulation	PTEN, BIM	oncomiR; inhibits apoptosis	[46]
miR-20a	upregulation	PTEN, BIM, PHF6	oncomiR; inhibits apoptosis	[46]
miR-20b	ETP-ALL low	−	ETP-ALL low	[32]
miR-21	upregulation	PDCD4	promotes survival	[50]
miR-29a	downregulation	−	negatively correlates with DFS	[84]
miR-29	downregulation	HBP1	tumor suppressor in NOTCH1-driven T-ALL model	[43]
miR-92	upregulation	IKAROS/IKZF1, PTEN, FBXW7, BIM, NF1	oncomiR; induces NOTCH1 expression; inhibits apoptosis	[46]
miR-128-3p	−	PHF6	oncomiR in a NOTCH1- driven T-ALL model	[49]
miR-151-3p	ETP-ALL low	−	ETP-ALL low	[32]
miR-193b-3p	downregulation	MYB	tumor suppressor in NOTCH1-driven T-ALL model	[42]
miR-200	downregulation	MYB, HBP1	tumor suppressor in NOTCH1-driven T-ALL model	[43]
miR-221	ETP-ALL high	−	ETP-ALL high; an independent predictive factor for shorter overall survival	[32, 83]
miR-222	ETP-ALL high	ETS1	ETP-ALL high; inhibits proliferation and causes apoptosis	[32]
miR-342-3p	ETP-ALL low	−	ETP-ALL low	[32]
miR-363	ETP-ALL low	−	ETP-ALL low	[32]
miR-576-3p	ETP-ALL low	−	ETP-ALL low	[32]
miR-590	upregulation	RB1	promotes proliferation, migration, and invasion	[29]
miR-664	upregulation	PLP2	promotes proliferation, migration, and invasion	[75]

### MiRNAs with other oncogenic signals in T-ALL

In addition to the well-known oncogenic NOTCH signaling pathway, there are many other biologically relevant pathways controlling cell growth, proliferation and survival, such as PI3K/AKT [44], NF-κB [45, 48], cAMP/PKA [57], and JAK/STAT pathway [58]; all of whose interactions with miRNAs also play important roles in T-ALL pathology.

As mentioned above, miR-19, which belongs to the miR-17-92 cluster, controlled multiple regulators (*PP2A*, *PRKAA1*, *BIM*, and *PTEN*) of PI3K signaling which resulted in increased phosphorylation of AKT and the ribosomal S6 protein, which subsequently promoted survival of T-ALL cells [44]. Additionally, miR-181a could induce chemoresistance in Jurkat T-ALL cells through activating AKT, which will be discussed in detail later [59]. Moreover, luciferase assay experiments showed that miR-19 directly repressed the expression of *CYLD*, which plays a predominant role in the negative regulation of NF-κB, inducing activation of the NF-κB downstream program [45]. Furthermore, miR-223 was transcriptionally activated by NF-κB signaling pathway [48]. Together, this research demonstrates that, the miRNAs and the NF-κB signaling pathway form a feedback regulatory loop.

MiR-142-3p has been shown to be highly expressed in human T-leukemic cells and has been correlated with poor prognosis of T-cell leukemia patients. MiR-142-3p acted in an oncogenic role in T-ALL by promoting leukemic cell growth and inducing glucocorticoid (GC) resistance through targeting both glucocorticoid receptor alpha (GRa) and cyclic adenosine monophosphate (cAMP)/protein kinase A (PKA) pathways [57]. MiR-21 has been identified as an oncogenic miRNA involved in NOTCH-mediated induction of T-ALL [50]. Recently it was also found upregulated in Jurkat cells, a T-ALL cell line, promoting cell proliferation, invasion, and decreased the apoptosis rate through the inhibition of signal transducer and activator of transcription 3 (STAT3) expression at the protein level [58].

### MiRNAs expression and transcription factors in T-ALL

In T-ALL development, the transcriptional program is strictly regulated. Pathological development of T-ALL requires aberrant expression of intact master developmental regulatory transcription factors that can function as oncogenes. One of these oncogenic transcription factors is *TAL1*/*SCL*, a member of basic helix-loop-helix (bHLH) family, which was aberrantly expressed in 60% of human T-ALL cases [60]. *TAL1*/*SCL* has been shown to initiate T-ALL in murine models [61, 62]. MiRNAs directly controlled by TAL1 were investigated via global miRNA expression profiling after depletion of *TAL1* and genome-wide analysis of *TAL1* occupancy by ChIP coupled to parallel DNA sequencing [47]. In this study, miR-223 was identified as the most upregulated miRNA by TAL1, and ChIP-sequence revealed that miR-223 was a direct target of TAL1. It was also shown that the expression of *TAL1* and miR-223 was closely correlated during normal T-cell development as well as T-ALL, with high expression in early thymocytes and marked downregulation after the double-negative-2 stage of maturation. Moreover, it was confirmed that miR-223 mediated overexpression of TAL1-induced growth of T-ALL cells through the direct inhibition of the expression of tumor suppressor, *FBXW7*. *FBXW7* has been shown to be able to repress *MYC*, *MYB*, *NOTCH1*, and *CYCLIN E* expression. Furthermore, another study of TAL1-regulated miRNAs in T-ALLs also showed the direct activation of miR-223, and inversely, the direct repression of miR-146b-5p by TAL1 [63]. Although transcription factor TAL1 is characterized as an oncogene during T-ALL progression, its aberrant activation is attributed to chromosomal rearrangement. However, there are some T-ALL patients who lack the *TAL1* locus rearrangement but have TAL1 activation. Unfortunately, to date the underlying mechanisms around this anomaly are still largely unknown. The same group further investigated whether epigenetic regulation of *TAL1* by specific miRNAs that may contribute to the ectopic expression of *TAL1* in some T-ALL cases [55]. By performing computational target prediction, luciferase reporter system, and mutagenesis assays; five candidate miRNAs (miR-101, miR-520d-5p, miR-140-5p, miR-448 and miR-485-5p) were found to directly target *TAL1.* Of these five targets, four miRNAs (miR-101, miR-140-5p, miR-448 and miR-485-5p) were downregulated in T-ALL patient specimens and T-ALL cell lines [55].

MiR-204 expression was decreased in T-leukemic cells compared to normal T-cell samples. Ectopic expression of miR-204 could inhibit proliferation, migration and invasion of T-ALL cells, indicating that miR-204 may act as a tumor suppressor in the regulation of cell growth and metastasis in T-ALL cells [64]. Moreover, the sex determining region Y-box 4 (SOX4), a transcription factor in development which belongs to the C subgroup of SRY-related HMG box (SOX) transcription factor family [65], was a direct target of miR-204. Its inactivation could partially mediate the tumor suppressor role of miR-204 in T-ALL [64]. On the contrary, miR-149^*^, acting as an oncomiR, was highly expressed in T-ALL cell lines and T-ALL patients’ bone marrow samples. MiR-149^*^ could promote proliferation and reduce apoptosis in T-ALL cells by directly targeting *JunB*, a transcription factor involved in regulating gene activity following the primary growth factor response [30]. Another example is the negative correlation between miR-181a and the early growth response 1 (EGR1) [66]. EGR1 is a member of the *EGR* gene family encoding for C2H2-type zinc-finger transcription factors [67], acting as a tumor suppressor to promote G0/G1 cell-cycle arrest [68]. Over-expression of miR-181a in Jurkat T-ALL cells could decrease *EGR1* expression, increase cell proliferation and enhance the cell-cycle progression from G0/G1 to S phase [66]. Dual luciferase assays revealed that miR-181a directly targeted *EGR1* followed by downregulation of *TGF1*, *BCL2*, *p53* and *p73*, whose transcription were promoted by EGR1 [69–74]. Strikingly, the rescue of EGR1 protein could revert the phenotype originally observed in Jurkat-miR-181a-overexpressing cells, suggesting that miR-181a behaved as an oncomiR in T-ALL by downregulating *EGR1*.

### MiRNAs expression and cell biological behavior deregulation in T-ALL

Aberrant expression of miRNAs has been implicated in the deregulation of various important cellular functions such as cell growth, apoptosis, migration, and invasion. All of these factors might contribute to or block the progression of leukemogenesis in T-ALL. As mentioned above, many miRNAs are involved in cell survival regulation during T-ALL progression via modulating key survival molecules or signaling pathways. The miR-17-92 cluster could suppress apoptosis and enhance survival of leukemic T-cells through decreasing E2F1 protein expression [34]. MiR-19 activated the PI3K/AKT pro-survival pathway via downregulation of several negative regulators [44]. MiR-142-3p targets the cAMP/PKA pathway to promote leukemic T-cell proliferation [57]. The oncomiR miR-21 was shown to promote cell proliferation, invasion, as well as decrease the apoptosis rate in Jurkat cells [58]. MiR-149^*^ was also classified as an oncomiR that promoted cell proliferation and suppressed apoptosis by targeting *JunB* in T-ALL cells [30]. Overexpression of miR-181a in Jurkat T-ALL cells promoted proliferation and enhanced the cell-cycle transition from G0/G1 to S phase through direct targeting of the transcription factor *EGR1* [66]. Moreover, miR-664 could promote proliferation, migration and invasion of T-ALL cells; and negatively regulate *PLP2* expression through direct binding of its 3′UTR [75]. MiR-590 was shown to inhibit *RB1* expression and promote proliferation and invasion of T-ALL cells [29]. In this study, miR-590 was found to be highly expressed in T-ALL samples as compared with normal healthy T-cell controls. It was shown to be negatively correlated with RB1 [76], a negative regulator of cell cycle, which was confirmed in 395 T-ALL patients samples. Overexpression of miR-590 promoted T-ALL cell proliferation by increasing G1/S transition, enhancing migration and invasion. Further studies revealed that miR-590 directly targeted and negatively regulated *RB1* in T-ALL cells, indicating that repressed *RB1* might mediate the biological effects of miR-590 on T-ALL cells.

On the contrary, there are many tumor-suppressive miRNAs that function as T-ALL progression inhibitors by suppressing cell proliferation, inducing cell apoptosis, and impeding cell migration and invasion capabilities. Overexpression of miR-222 inhibited proliferation and caused cell cycle arrest and apoptosis in leukemic cells by directly suppressing *ETS1* expression [32]. Enforced expression of a set of tumor suppressor miRNAs (miR-29, miR-31, miR-150, miR-155, and miR-200) had anti-proliferative effects in four human T-ALL cell lines [43]. MiR-101 repressed proliferation and invasion, and induced apoptosis in Jurkat cells by direct downregulation of *NOTCH1* expression [19]. MiR-204 was decreased in T-ALL, and its ectopic expression suppressed cell proliferation, migration and invasion [64]. Overexpression of the tumor suppressive miRNA miR-146b-5p was confirmed to repress the *in vitro* migration and invasion of T-ALL cells, and extend mouse survival in a xenotransplantation model of human T-ALL *in vivo* [77].

### MiRNAs expression and drug resistance and prognosis in T-ALL

Leukemia drug resistance is a particularly troubling challenge in T-ALL, and is associated with relapse and dismal prognosis despite intensified chemotherapy [78, 79]. This leads to a considerable proportion of patients with T-ALL who fail to obtain complete remission and eventually die as a result of disease progression [80, 81]. Epigenetic studies have started to reveal the relevance between chemoresistance, prognosis and deregulation of miRNAs in T-ALL; however, much efforts remain to be devoted to research in these areas.

MiR-142-3p was upregulated in human T-leukemic cell lines and primary T-leukemic cells, and could induce glucocorticoid resistance by decreasing GRa protein expression through directly targeting the 3′UTR of *GRa* mRNA. The suppression of the *GRa* was concomitant with the higher expression of miR-142-3p in T-ALL patients with poor prognosis as compared to those with good prognosis [57]. The high expression of miR-181a in T-ALLs was associated with multi-drug resistance through the activation of the AKT signaling pathway [59]. In this study, overexpression of miR-181a in the Jurkat cell line could increase AKT phosphorylation. MiR-181a and phosphorylated AKT were significantly increased when Jurkat cells exposed to chemotherapeutic agents, including doxorubicin (DOX), cyclophosphamide (CTX), cytarabine (Ara-C), and cisplatin for 48 h. Specific inhibition of miR-181a in Jurkat cells and its resistant sublines increased cell sensitivity to DOX, in accordance with decreased AKT phosphorylation; and decreased miR-181a expression was also related to enhanced cell sensitivity to cisplatin and CTX in doxorubicin-resistant Jurkat cells [59]. In contrast, the tumor suppressive miRNA, miR-101 was reported to enhance drug sensitivity of Jurkat cells [19]. In this study, miR-101 expression was significantly decreased in Jurkat cells following treatment with Adriamycin.

The association of distinct miRNAs expression in relation to risk categories in T-ALL has been investigated in a number of publications. High miR-16 expression was found to be associated with hyperleukocytosis and poor cytogenetic groups in 93 childhood ALL cases [82]. In T-ALL cases, a significant trend was found between the shortening of a patient's survival rate associated with the lowest to the highest miR-16 levels for both disease-free survival (DFS) and overall survival [82]. MiR-221 was highly expressed in T-ALL samples from 48 T-ALL patients and was an independent predictive factor for shorter overall survival [83]. Oliveira *et al*. found that miR-29a expression level was extremely reduced in T-ALL cells compared to normal T-cells, and lower level of miR-29a is associated with higher blast counts in the bone marrow and increased DFS in T-ALL patients [84].

## CONCLUSIONS

The identification and molecular characterization of new oncogenic and tumor suppressive miRNAs in T-ALL have provided new insights into the pathogenesis of this disease. Since miRNA regulates or can be regulated by multiple genes and signaling pathways that affect various cell functions, it can be used as markers for disease diagnosis, progression, treatment response and clinical outcome [32, 51, 57, 84]. A number of key signaling pathways that play critical role in T-ALL pathology and progression are involved in the miRNA regulatory network including the NOTCH pathway, whose aberrant constitutive activation is believed to be the predominant oncogenic event in the pathogenesis of T-ALL [13]. With NOTCH1-driven T-ALL mouse model, many miRNAs with oncogenic or tumor suppressive roles in T-ALL onset and progression have been elucidated (Figure 1). Moreover, miRNA expression profiles, target prediction algorithms of miRNAs, and key protein-coding genes known to be involved in T-ALL, as well as functional studies have created a complicated co-regulatory network that eventually influence leukemia pathogenesis, progression, treatment, and prognosis (Table 1).

MiRNAs might be better therapeutic targets because their expression are often tissue specific. Additionally research has shown that there is one specific subtype of miRNA that plays different roles during normal thymic T-cell development and leukemogenesis. For example, in a study of examination of the roles of three *mir-181* genes (*mir-181ab1*, *mir-181ab2* and *mir-181cd*) in normal thymocyte development and in T-ALL development [56], it was found that deletion of *mir-181ab1*, but not *mir-181ab2* and *mir-181cd*, effectively inhibited NOTCH1-induced T-ALL [56]. More important, the effects of *mir-181ab1* deletion were compensated for during normal thymic progenitor development but not during T-ALL development. As a result, deletion of *mir-181ab1* gene could specifically inhibit the activity of the *NOTCH* oncogene in tumorigenesis without causing significant defects in normal development [56]. Thus, drugs targeting these tissue specific miRNAs could be less off-target and cause less toxicity to normal tissues. Better understanding of the co-regulatory molecular mechanism between miRNAs and protein coding genes involved in T-ALL will contribute to more targeted-therapies for patients. Although the delivery efficiency remains challenging, therapies with small interfering RNAs and antisense oligonucleotides or a miRNA mimic targeting specific miRNA could show promising prospects with technical improvements in the future.
